# The cell cycle as a brake for β-cell regeneration from embryonic stem cells

**DOI:** 10.1186/s13287-015-0274-z

**Published:** 2016-01-13

**Authors:** Ahmed El-Badawy, Nagwa El-Badri

**Affiliations:** Center of Excellence for Stem Cells and Regenerative Medicine (CESC), Zewail City of Science and Technology, Sheikh Zayed District, 12588 6th of October City, Giza Egypt

## Abstract

The generation of insulin-producing β cells from stem cells in vitro provides a promising source of cells for cell transplantation therapy in diabetes. However, insulin-producing cells generated from human stem cells show deficiency in many functional characteristics compared with pancreatic β cells. Recent reports have shown molecular ties between the cell cycle and the differentiation mechanism of embryonic stem (ES) cells, assuming that cell fate decisions are controlled by the cell cycle machinery. Both β cells and ES cells possess unique cell cycle machinery yet with significant contrasts. In this review, we compare the cell cycle control mechanisms in both ES cells and β cells, and highlight the fundamental differences between pluripotent cells of embryonic origin and differentiated β cells. Through critical analysis of the differences of the cell cycle between these two cell types, we propose that the cell cycle of ES cells may act as a brake for β-cell regeneration. Based on these differences, we discuss the potential of modulating the cell cycle of ES cells for the large-scale generation of functionally mature β cells in vitro. Further understanding of the factors that modulate the ES cell cycle will lead to new approaches to enhance the production of functional mature insulin-producing cells, and yield a reliable system to generate bona fide β cells in vitro.

## Background

Stem cells are characterized by their prominent capacity to self-renew and to differentiate into multiple lineages of cells. Stem cell therapy has the potential to treat intractable disease and to be applied for tissue engineering and drug screening. Recent strategies in stem cell research have succeeded in generating differentiated cells that are otherwise hard to replace [1]. These cells have been transplanted into animal models with promising results [2]. One of the rapidly growing diseases that may be treatable by stem cell therapy is diabetes mellitus (DM), which affects more than 300 million individuals worldwide according to the International Diabetes Federation [3]. Type 1 DM results from autoimmune destruction of β cells in the pancreatic islets, whereas the more common type 2 DM results from peripheral tissue resistance to insulin, and subsequent β cell dysfunction.

Development of cell therapy for type 1 DM has shown some success following the Edmonton protocol, in which diseased islets are replaced by healthy ones from cadaveric donors [4]. This procedure, however, suffers many challenges—especially the limited supplies of islets and their high variability—caused by donor genetic background and other factors in their isolation [5]. A single 68 kg (150 lb) patient, for example, requires roughly 340–750 million transplanted islet cells to effectively resolve type 1 DM [6–8]. In clinical practice, this requires two or three donors of pancreatic islets for a transplantation procedure into a single patient. Therefore, the generation of a sufficiently large supply of human β cells from the same patient’s stem cells could extend stem cell therapy to millions of new patients suffering from DM. Additionally, genetically diverse stem cell-derived β cells could be used for disease modeling either in vitro or in vivo.

The maintenance of β-cell number and islet mass is essential to maintaining normoglycemia [9]. In fact, the production of these insulin-producing cells in adults often occurs through self-duplication of mature cells instead of differentiation of their stem-cell progenitors [10–12]. Regardless of the signals required to stimulate β-cell regeneration, they must all act on the basic cell cycle replicative machinery. Therefore, analyzing the pathways that control β-cell regeneration could allow for novel interventions to introduce a radically new dynamic to the field of β-cell regeneration. Here, we present perspective on the molecular mechanisms that control cell cycle regulation during β-cell regeneration, and consider the potential application of cell cycle modulation for large-scale production of functional β cells from embryonic stem (ES) cells as an effective approach for treatment of DM.

Since the process of stem cell differentiation requires the coordination of cell cycle progression and cell fate choices [13–15], we discuss the cell cycle control mechanisms in ES cells and β cells in the first part of this review. We then highlight the fundamental differences between pluripotent cells of embryonic origin and differentiated β cells. Based on these differences, we propose that ES cells do not adopt the proper cell cycle machinery for β-cell regeneration. Modulation of this unique cell cycle machinery presents a unique target to develop novel strategies to produce large numbers of functionally mature insulin-producing cells in vitro.

## The cell cycle of ES cells and pancreatic β cells: uniqueness and divergence

The use of stem cells in the generation of a renewable source of β cells remains a realistic promise. However, many issues still need to be resolved before this strategy becomes a practical therapeutic option. Although ES cells seem to have the highest potential to differentiate into insulin-secreting cells [16], one of the main limitations is the lack of responsiveness to glucose stimulation [17, 18]. Recent studies have shown, however, that pancreatic endoderm cells derived from human ES (hES) cells can produce insulin in response to glucose several months after transplantation into immunodeficient mice [19].

### Cell cycle regulation in ES cells

Pluripotent cells in the epiblast were shown to have a cell cycle profile that lacks fully developed G1 and G2 gap phases, in which a longer time (approximately 60 %) is allocated to the S phase. A similar cell cycle structure has been described in ES cells [20, 21], which have an unusual cell cycle structure comprising mainly S phase cells and a truncated G1 phase [22]. Interestingly, lengthening the G1 phase by manipulating cell cycle regulators is sufficient to drive differentiation [23], suggesting that G1 lengthening is a cause rather than a consequence of differentiation. In hES cells, the cells reside in S phase 65 % of the time, and in G1 phase only 15 % of the time [24]. Also, the cell cycle of induced pluripotent stem cells has shown a similar pattern, indicating that rapid division and shortening of the cell cycle may be crucial for pluripotency [25–27].

The cell cycle represents a tightly regulated process of cell replication and cell division, and has an important role in regulation of cell fate decision. The hypothesis that a link exists between cell cycle regulation and cell fate decisions is supported by the observation that the essential pluripotency factors OCT4, SOX2, and NANOG control the expression of key cell cycle regulatory proteins such as CDK1, cyclin D1, CDK6, CDC7, and CDC25A [28, 29]. Evidence for another link between cell cycle regulation and self-renewal machinery came from a study by Chavez and colleagues [30], where E2F was shown to be a possible regulatory cofactor for OCT4 [30]. Many additional networks are likely to exist between key cell cycle regulators and stemness, including control via microRNAs [31]. For instance, the c-Myc/E2F-driven miR-17-92 cluster, which controls the G1–S transition, is fundamental for hES cell self-renewal and cell proliferation and is decreased upon hES cell differentiation [32]. Furthermore, recent studies have shown that cell cycle regulation is a rate-limiting step in the process of reprogramming somatic cells [33–35]. The reprogramming factor KLF4 was found to likely function by suppression of p53 [36]. Another factor, LIN28, a positive regulator of cyclin A, cyclin B, and CDK4 [37], was found to improve reprogramming efficiency [38] by accelerating cell division [39]. Together these studies show a strong possibility of reprogramming somatic cells by inducing ES cell-specific cell cycle characteristics. Furthermore, a direct relationship between cell cycle regulation and pluripotency of ES cells has been shown, by linking Oct4-regulating genes to cell cycle progression [40]. Inhibition of Oct4 prompted downregulation of genes involved in ES cell proliferation, and an upregulation in the cell cycle inhibitor p21 and expression of p63, all of which have been linked to differentiation [41].

Although Cdk2 is considered the principal Cdk in hES cells [20, 42], Cdk4–cyclin D2 complexes have shown little activity in murine ES (mES) cells [22, 25, 43]. Rapid progression of the cell cycle in mES cells was shown to be due to an unusually high Cdk2 activity in undifferentiated ES cells which is not under the control of the cell cycle [20]. This high level of Cdk2 activity is due to the continuous expression of both cyclin E and cyclin A during the cell cycle of mES cells [20]. Predictably, inhibition of Cdk2 activity with the Cdk2 inhibitor Olomoucine II was shown to restrain mES cell proliferation by keeping the cells in the G1 phase and preventing G1–S transition without affecting ES cell pluripotency [44].

In hES cells, knockdown of CDK2 activity was shown to delay the G1–S transition and cause G1 arrest [45, 46]. Similarly, CDK2 activity was also crucial for cell fate decisions in hES cells [46, 47]. Similar to mES cells, hES cells do not express CDK inhibitory proteins of the Ink4 and Cip/Kip families [22, 48]; therefore, CDKs have very high activity, with CDK2 showing the overall highest kinase activity in hES cells [45], indicating a critical role for CDK2 in G1 phase regulation in both mES cells and hES cells.

Filipczyk et al. [49] showed that hES cells do not express any of the D-type cyclins. Moreover, they demonstrated that 100 % of hES cells were positive for cyclin E1 protein, suggesting that cyclin E1 is substantially expressed in undifferentiated hES cells, as is the case with mES cells [50, 51]. Neganova et al. [45] compared the expression of all major cyclins in undifferentiated hES cells and in hES cells that were differentiated into embryoid bodies. They showed that cyclins E1, A2, and B1 vacillated during the cell cycle, and undifferentiated hES cells expressed only cyclins D1 and D3, while the level of cyclin D2 was very low or undetectable [45]. Cyclin E on the other hand was shown to be involved in maintaining the pluripotent state of ES cells [50, 51]. Overexpression of cyclin E supported ES cell self-renewal, and increased the resistance of ES cells to transient leukemia inhibitory factor withdrawal. However, loss of cyclin E1 expression in ES cells elongated G1 phase and induced differentiation [52].

### Cell cycle regulation in pancreatic β cells

The replication of β cells is regulated by a network of tightly orchestrated cell cycle regulators. The expression of these regulators corresponds to the ability of β cells to replicate at different life stages [53–56]. Cell cycle progression in murine islets is controlled by three types of D cyclins (D1, D2 and D3), which bind to and activate CDK4. As molecular oncologists interested in overall cell replication, these investigators generated mice that lack Cdk-4, and predicted a generalized decline in cell proliferation rates. Interestingly, the data revealed that global Cdk-4 deletion led to a very restricted phenotype as Cdk-4 knockout mice showed abnormalities in only three tissues—ovary, testis, and pancreatic β cells [57, 58]. Pancreatic islets showed cell hypoplasia, which resulted in diabetes and ketoacidosis [58]. These findings would seem to indicate a marked specificity of the effect of the Cdk-4–cyclin D pathway on the cell cycle in β cells, but they fail to demonstrate why or how it is so tissue-specific [58]. Furthermore, the adenoviral delivery of CDK4 into human and rat β cells did increase β-cell proliferation rates, which were even further increased by cyclin D1 [59]. Moreover, Sertad1 (Sei1), which helps stabilize the CDK4–cyclin D complex by hindering p16 binding, thereby advancing cell cycle progression [60], is expressed at significantly high levels in pancreatic islets. In fact, Sei1-null mice present mild defects in the islets [60]. Lastly, after partial pancreatectomy, mice expressing an active Cdk4^R24C/R24C^ kinase had higher β-cell mass compared with wild type due to elevated levels of β-cell proliferation in the mutant mice, though not necessarily at an accelerated pace [61]. Taken together, these studies show that regulation of Cdk4 is critical to the maintenance of β-cell replication.

Cyclin D1 and D2 are expressed in pancreatic islets, the latter at higher levels [62]. As β-cell replication diminishes with age, so do the expression levels of cyclin D1 and D2 [63]. Cyclin D2, although not needed for neonatal development, is critical for controlling β-cell growth and replication [62]. Cyclin D1 partially compensates for the absence of cyclin D2, since the double mutant aggravates this phenotype, with uncontrollable diabetes leading to death at an early age [62]. These studies highlight the importance of cyclin D in regulating and controlling β-cell proliferation and islet mass. Consistent with the concept that increased cyclin D expression causes higher replication rates, adenoviral-mediated expression of cyclin D1 in both human and murine islet cells causes increased proliferation [59]. Subsequent to this finding, Daniel Chung and colleagues [64] overexpressed cyclin D1 in murine islets in vivo and showed an exceptional increase in islet mass without hypoglycemia, proposing β-cell replication as the cause of the islet hyperplasia. Interestingly, the β-cell-specific overexpression of cyclin D1 did not cause hypoglycemia; in fact, glucose and insulin levels remained similar to those in the wild type [64]. Collectively, these studies provide direct evidence that deletion of cyclin D from the murine islet stops the cell cycle, diminishing β-cell proliferation rates. Meanwhile, overexpressing cyclin Ds in β cells causes increased proliferation through overactive cell cycle progression.

### Comparison of the cell cycle in ES cells and β cells

**Fig. 1:**
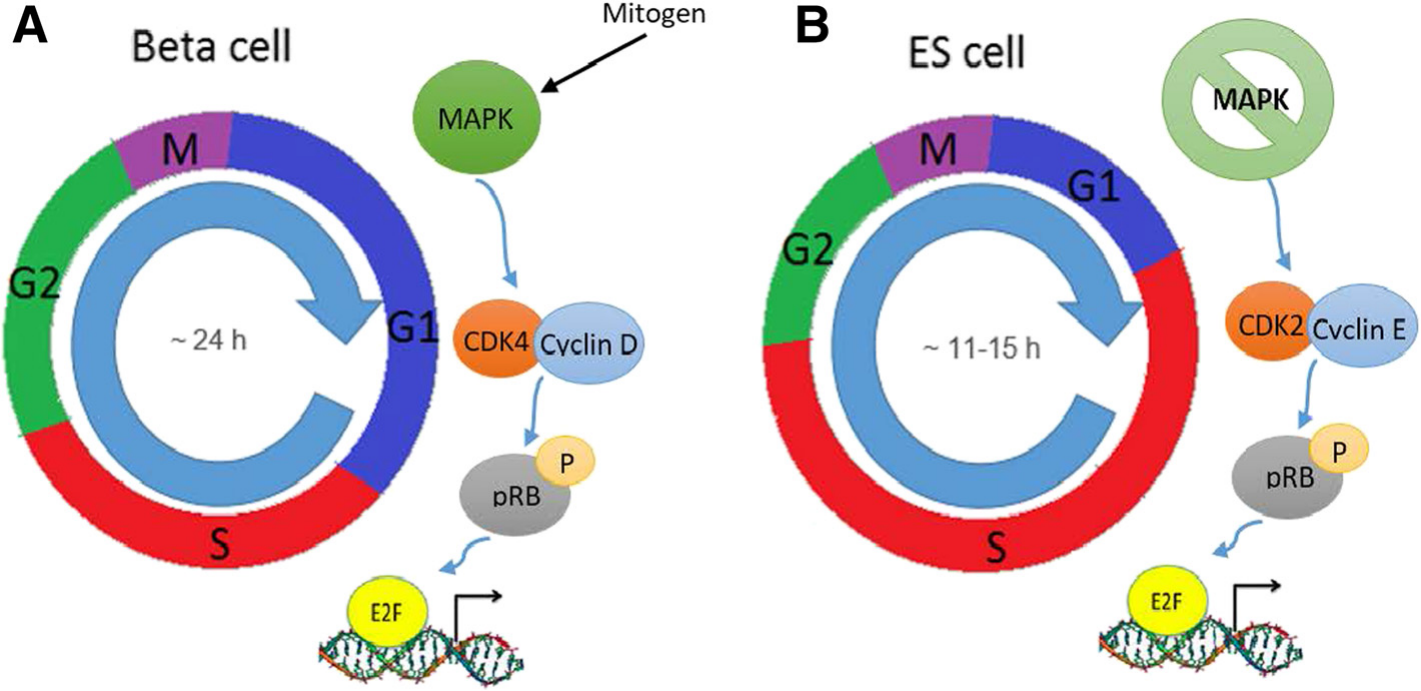
Differences in the cell cycle machinery of β cells (**a**) and undifferentiated embryonic stem (*ES*) cells (**b**). The cell cycle of ES cells is shortened relative to that of β cells. An abbreviated G1 phase is responsible for the difference in cell cycle length. **a** In β cells, mitogen signaling through the mitogen-activated protein kinase (*MAPK*) pathway activates CDK4–cyclin D kinase activity, thus hypophosphorylating retinoblastoma protein (*pRB*), which then leads to release of E2F, allowing it to activate transcription of genes necessary in the progression of the cell cycle. **b** In ES cells, mitogen signaling through the MAPK pathway seems to be irrelevant in the progression of cell cycle. Cyclin E–CDK2 expression is cell cycle-independent and constitutively active throughout the cell cycle, which allows the transition of ES cells from M phase directly to late G1. The resulting absence of the cyclin D-dependent early G1 phase shortens the G1 phase and the entire cell cycle. In beta cells, cyclin D–CDK4 is highly active but cyclin E–CDK2 is absent, making the cell cycle between these two cells highly different

Both ES cells and β cells have unique cell cycle machineries that exhibit significant differences from each other (Table 1). ES cells have an unusual cell cycle, comprising mainly an S phase and a short G1 phase [22] (Fig. 1). Regarding the CDKs, Cdk4–cyclin D2 complexes have very limited activity in ES cells [22, 25, 43], where Cdk2 is considered the principal Cdk [20, 42]. Interestingly, Cdk4 is highly expressed in β cells and is not just an important regulator for cell cycle progression but is also critical for β-cell development [57, 58]. However, Cdk2 has very limited activity in β cells.

**Table 1:** Differences between the cell cycle machinery of β cells and ES cells

Cell cycle machinery	Beta cells	ES cells	References
Cdk2	Expressed but no functional importance	Critical for ES cell maintenance and considered the primary Cdk	[20, 42, 85]
Cdk4	Critical for β-cell development	Not expressed in ES cells	[22, 25, 43, 57, 58]
Cdk6	Not expressed	Expressed at high levels	[22, 86]
Cyclin D1	Expressed at high levels	Expressed at low levels	[62]
Cyclin D2	Critical for development and highly expressed	Not expressed in ES cells	[62, 63]
Cyclin E	Expressed but no importance	Critical for maintaining the pluripotent state of ES cells	[50, 51, 87, 88]

*ES* embryonic stem

**Fig. 2:**
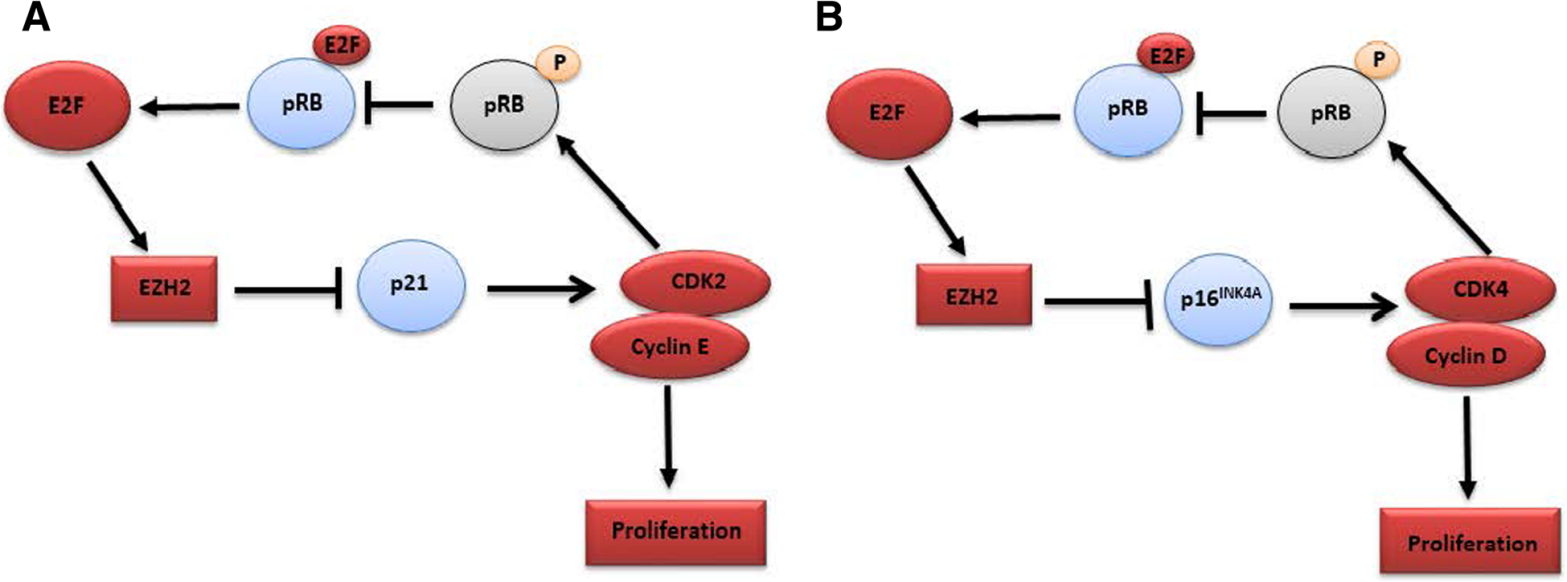
Cell cycle regulation of ES cells (**a**) and β cells (**b**). Upregulated components are indicated in *red* and inhibited proteins are indicated in *blue*. Levels of the Polycomb protein enhancer of zeste homologue 2 (*EZH2*) are increased during cell proliferation, which leads to a decrease in levels of the cell cycle inhibitors p21 and p16^INK4A^. **a** In ES cells, Cdk2–cyclin E is considered the primary cell cycle complex and, once activated, it phosphorylates retinoblastoma protein (*pRB*). Once pRB has been phosphorylated, E2F is released. The released E2F is then free to act as a transcription factor and it subsequently binds to DNA promoter regions and activates the expression of proteins required in the next stages of the cell cycle and in DNA replication. **b** In β cells, Cdk4–cyclin D is considered the primary cell cycle complex and is responsible for pRb phosphorylation

Regarding the cyclins, it has been shown that ES cells do not express any of the D-type cyclins [49]. However, 100 % of hES cells were positive for cyclin E1 protein, which is presumably constitutively expressed in both undifferentiated hES cells and mES cells [50, 51]. In contrast to other cell types where D-type cyclin-dependent kinases are not essential for cell cycle entry [65], cyclins D1 and D2 are critical for β-cell development, where they are highly expressed in G1 phase. They control the activity of CDK4/6 that inhibits retinoblastoma protein (pRB) phosphorylation and frees E2F to start the G1–S transition. In contrast, cyclin Ds are expressed at low levels in ES cells with very low activity of CDK4, whereas pRB is constitutively phosphorylated by CDK2–cyclin E, bypassing the need of a G1 checkpoint (Fig. 2). Furthermore, the pluripotency of ES cells relies on CDK2 activity.

Therefore, the Cdk4–cyclin D2 complex that is critical for β-cell development and replication has very limited activity in ES cells, with undetectable levels of cyclin D2. It is thus highly likely that the unique cell cycle machinery of ES cells does not support the generation of β cells, which may provide a plausible explanation for the difficulty in generating functional β cells from them in large quantities.

## Do ES cells adopt the proper cell cycle machinery for β-cell differentiation?

Murine studies demonstrated that cyclin Ds are expressed in a tissue-specific manner during gastrulation in the mouse embryo. For example, the mesoderm expresses cyclin D1/D2, the neuroectoderm expresses cyclin D1/D2, and the endoderm expresses a low level of cyclin D2, whereas cyclin D3 is specifically expressed in the trophectoderm. These studies provided the first evidence for an interconnection between cell fate decision and cell cycle regulation. Growth and maintenance of adult β-cell mass represents a unique model for G1 cell cycle biology. The expansion of β-cell mass in adults seems to be crucially dependent on mitogenic signals acting via D-type cyclin/Cdk4 activity (Fig. 1) [66]. A clear observation of this phenomenon was shown when the whole body deletion of Cdk4 in mice specifically restricted β cell proliferation [58]. It has been shown that one of the D-type cyclins, cyclin D2, is uniquely required for β-cell replication and the proper replication of β-cell mass during postnatal development. Cyclin D2 thus seems to have a critical role within β cells in mediating mitogenic stimuli to control β-cell mass in the pancreas [67].

Attempts at developing insulin-producing cells from hES cells in vitro have only succeeded, to date, in generating cells with abnormal or immature phenotypes [68]. For example, these cells either fail to respond to glucose-induced insulin expression in vitro or fail to express appropriate β-cell markers, such as NKX6-1 or PDX1. They also abnormally coexpress different hormones, such as glucagon along with insulin, fail to function after transplantation in vivo, or show a combination of these abnormal properties [69–73]. These abnormalities may be accounted for by the unique physiological cell cycle machinery of ES cells, which do not adopt the proper cell cycle mechanisms necessary for generating functionally mature β cells. Of special importance is the Cdk4–cyclin D complex, which is critical for β-cell generation, and is not present in ES cells.

The Cdk4–cyclin D2 pathway has been suggested to have different roles in β-cell function, in addition to the control of cell proliferation, since it is robustly expressed in non-proliferating β cells [74]. First, Cdk4 directly regulates expression of Kir6.2, which is a key component of the K_ATP_ channel involved in the regulation of glucose-induced insulin secretion [74]. Consistently, inhibition or genetic inactivation of CDK4 results in decreased expression of Kir6.2, impaired insulin secretion and glucose intolerance in mice [74]. Second, Cdk4 is essential in regulating early pancreas development and promotes β-cell mass expansion via activation of Pdx1 and Ngn3 expression [75]. Third, Cdk4 deficiency specifically reduces embryonic pancreas size owing to fewer Pdx1^+^ pancreatic progenitor cells. Additionally, the expression of activated Cdk4^R24C^ kinase leads to increased Nkx2.2^+^ and Nkx6.1^+^ cells and also leads to a rise in the number and proliferation of Ngn3^+^ endocrine precursors, resulting in expansion of the β-cell lineage [75]. Forth, Cdk4 catalyzes the recruitment of quiescent cells within the islets and the ductal epithelium to participate in the regenerative process [61]. Altogether, it is clear that while the Cdk4–cyclin D2 complex is critical for β-cell development and replication, it has very limited activity in ES cells, given their undetectable levels of cyclin D2. This may present a possible explanation for the failure to produce functional β cells from ES cells on a large scale. This can also explain the lengthy period of several months currently required for β-cell differentiation from ES cells.

While these differences in the cell cycle machinery between β cells and ES cells may constitute challenges to current stem cell differentiation protocols, they can also be viewed as opportunities for further studies in regulating the cell cycle machinery of ES cells to mimic the machinery required for large-scale production of functional β cells in vitro. However, more research is needed to determine the extent to which the cell cycle of ES cells might contribute to β-cell regeneration, and to establish a causal link between the cell cycle machinery and β-cell regeneration from ES cells.

## The potential of regulating the cell cycle of ES cells to ensure large-scale production of functional β cells

The generation of adult β cells takes place mostly through self-replication instead of differentiation from stem cell progenitors; therefore, the cell cycle plays a fundamental role in β-cell regeneration. In order to achieve large-scale production of functional β cells from ES cells, regulating the cell cycle machinery of ES cells seems to be a worthwhile direction. Since β cells are now known to be among the most slowly replicating cells in the body, and because of their unique cell cycle machinery, special modulation of ES cells may be necessary to achieve effective differentiation.

Pauklin and Vallier [14] have shown that the cell fate decision is tightly linked to the cell cycle machinery, and proposed a few mechanisms that synchronize differentiation and proliferation in developing tissues. They also demonstrated that cell cycle manipulation using a small molecule directs differentiation of human pluripotent stem cells, and thus provides an approach to generate cell types of clinical interest. This led to the suggestion that simple manipulation of the cell cycle using small molecules could direct differentiation of pluripotent stem cells towards particular cell types without the need for exogenous growth factors [14].

**Fig. 3:**
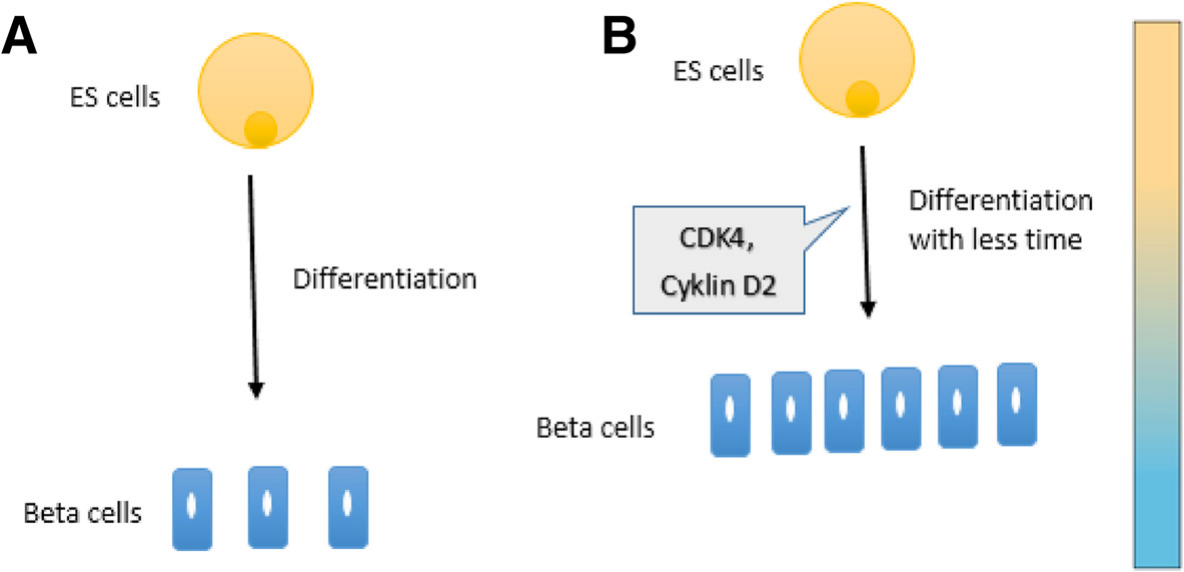
Proposed differentiation models. **a** Embryonic stem (*ES*) cells can differentiate into functional β cells within 3–4 months in small numbers. **b** Modulating the cell cycle machinery of ES cells by transient overexpression of the CDK4–cyclin D2 complex prior to β-cell differentiation protocols may seem to be an attractive direction for the large-scale generation of functionally mature β cells in less time

Remarkably, in a recent study by Chen et al. [76], complete regeneration of pancreatic islets and improvement of streptozotocin-induced diabetes in rats was achieved following gene therapy with Cdk4–cyclin D2 plasmids. The study reported that this plasmid gene therapy did not result in activation of oncogenes [76], and that β-cell regeneration was not mediated by self-replication of pre-existing β cells. Instead, Cdk4–cyclin D2 initiated active proliferation of adult pancreatic progenitor cells that existed within the islets [76]. Since the efficiency of ES cells to differentiate into tissue-specific cells is influenced by their cell cycle [14], modulating the cell cycle machinery of ES cells by transient overexpression of the Cdk4–cyclin D2 complex prior to β-cell differentiation protocols seems to be an attractive direction, and may open the gate for generating large numbers of functional β cells within a short time (Fig. 3).

Considerable work still remains to be done to completely understand the role of the cell cycle machinery of ES cells in their differentiation into functional β cells. A growing body of knowledge suggests that manipulation of the cell cycle and controlled regulation of its various phases may represent a fundamental and novel approach to the generation of large numbers of β cells. Developing novel strategies to precisely control progenitor cell differentiation will significantly benefit from studying the mechanisms that control the relationship between the ES cell cycle and β-cell regeneration. The time is ripe for such studies to leverage the growing knowledge about cell cycle regulation for translational research.

## Conclusion and perspectives

The generation of large numbers of β cells from hES cells was recently possible by using specified genes and signals that were based on studies of pancreatic development [77–79]. Endoderm and derived pancreatic progenitors can now be differentiated with high efficiencies [19, 70, 80]. These cells can differentiate into functional β cells within 3–4 months following transplantation into rats [19, 80], demonstrating that a few cells in the preparation contain the developmental capacity to generate β cells when given enough time and proper cues. Unfortunately, the reason why it takes several months for the cell differentiation in vivo is not fully understood. Furthermore, there is no evidence that comparable in vivo differentiation into β cells would apply to human patients. To date, approaches that include pancrease transplantation, islet cell transplantation, and administration of anti-CD3 monoclonal antibody have been approved for clinical therapy of DM [81–83]; however, these procedures suffer many challenges, especially the limited supply of islets and their high variability, caused by donor genetic background and other factors in their isolation [5]. This has lent strong impetus to the search for new sources of insulin-producing cells.

Both hES and mES cells have been reported to have cell cycle features that are more characteristic of somatic cycling cells [84]. Li et al. [13] showed that molecular pathways controlling the cell cycle could be engineered to basically affect ES cell differentiation at early stages in vitro. Strategies based on modulating these pathways can shorten the rate and simplify the lineage path of ES differentiation [13]. This makes it likely that pathways involving cell proliferation interact at different points with pathways that control cell lineages in embryos, and demonstrates that this knowledge can be used productively to guide the path and efficiency of cell differentiation of pluripotent cells.

Both β cells and ES cells have very unique cell cycle machineries but with a large difference. Cdk4 and its binding partner, cyclin D2, seem to be critical in β-cell development. In contrast, ES cells have very limited Cdk4–cyclin D2 activity, where Cdk2–cyclin E is considered the primary cell cycle complex. Accordingly, ES cells do not have the Cdk4 and the cyclin D2 that is critical for β-cell regeneration. This might be a possible explanation for the difficulty in generating functional β cells in large quantities from them.

Through critical analysis of the differences of the cell cycle between these two cells, we propose that the cell cycle of ES cells may act as a brake for β-cell regeneration from ES cells and that their unique cell cycle machinery may be a possible cause for the generation of β cells with abnormal or immature phenotypes. This may also explain the several month-long process of β cell differentiation from ES cells. It is possible, therefore, that the unique differences between the cell cycle machineries of β cells and ES cells constitute some form of barrier for generating functional mature β cells from ES cells. However, understanding these differences should lead to new strategies for modulating the cell cycle of ES cells to favor the generation of functional β cells.

Emerging data suggest that there are several links between basic cell cycle mechanisms and the capacity to drive ES cells to differentiate into a desired lineage; in this case, β cells. Based on our hypothesis that the cell cycle may act as a brake for β-cell regeneration from ES cells, we propose that modulating the cell cycle machinery of ES cells prior to β-cell differentiation protocols may present opportunities for the development of revolutionary therapies for diabetes and its complications.

## Abbreviations

DM: Diabetes mellitus
ES: Embryonic stem
hES: Human embryonic stem
mES: Murine embryonic stem
pRB: Retinoblastoma protein
